# Using a Developmental-Relational Approach to Understand the Impact of Interpersonal Violence in Women Who Struggle with Substance Use

**DOI:** 10.3390/ijerph16234861

**Published:** 2019-12-03

**Authors:** Naomi C. Z. Andrews, Mary Motz, Bianca C. Bondi, Margaret Leslie, Debra J. Pepler

**Affiliations:** 1 Department of Child and Youth Studies, Brock University,1812 Sir Isaac Brock Way, St. Catharines, ON L2S 3A1, Canada; 2 Mothercraft, Early Intervention Department, 860 Richmond Street West, Toronto, ON M6J 1C9, Canada; mmotz@mothercraft.org (M.M.); mleslie@mothercraft.org (M.L.); 3 Department of Psychology, York University, 4700 Keele Street, Toronto, ON M3J 1P3, Canada; bbondi@yorku.ca (B.C.B.); pepler@yorku.ca (D.J.P.)

**Keywords:** interpersonal violence, domestic violence, substance use, intervention, women, developmental-relational, gender-specific approach

## Abstract

Substance use among women is a major public health concern. This review article takes a developmental-relational approach to examine processes through which early relational trauma and violence in relationships may lead to substance use. We examine how early exposure to violence in relationships can impact neurological development, specifically through interference with physiological mechanisms (e.g., the hypothalamic-pituitary-adrenal axis), brain structure and functioning (e.g., the hippocampus and prefrontal cortex), and neuropsychological development (e.g., executive functioning and emotion regulation) across the lifespan. Further, we discuss the impact of exposure to violence on the development of relational capacity, including attachment, internal working models, and subsequent interpersonal relationships across the lifespan, and how these developmental pathways can lead to continued problematic substance use in women.

## 1. Introduction

Substance use among women is a major public health concern. There is recognition of the concurrent challenges associated with substance use and the barriers to reduce use for women (e.g., poverty, untreated mental health difficulties [1,2]). Another challenge is women’s experiences with interpersonal violence. Interpersonal violence is commonly thought of as domestic violence or intimate partner violence. Though both women and men experience violence in partner relationships, women are more often victims of violence in relationships, experience more severe forms of violence, and are more afraid of the harm that abusers cause than are men (e.g., [3]). Importantly, experiences with violence in relationships often begin before women enter adulthood (e.g., via childhood maltreatment, witnessing violence between parents) and continue throughout adolescence and into early adulthood. It is for this reason that we use the term interpersonal violence (IPV), to highlight the developmental and intergenerational nature of violence in relationships and that violence in relationships is not exclusive to violence between partners. For many women, the struggle with substance use arises from their experiences of trauma and violence in relationships across development. Though there are many types of trauma for women, we will focus on interpersonal trauma, or trauma associated with violence in relationships. In this review paper, we discuss developmental mechanisms—neurological and relational—through which early and ongoing experiences with IPV can lead to substance use. Though these developmental mechanisms may exist for both women and men, this paper will focus on what we know about these processes among women. That is, through clinical experience working with women who have substance use issues and a review of literature on women’s substance use and relationships, this paper will explore the ways in which substance use may be a mechanism for coping with negative and traumatic relational experiences that many women have experienced since childhood and across development. Further, given that women are more often victims of interpersonal violence than are men [3], we focus on the pathways to substance use for women using a developmental-relational approach, and describe the importance of gender-specific programming for women who have experienced IPV and who use substances.

## 2. A Developmental-Relational Approach to Understand the Impact of IPV for Women

A developmental-relational approach is one in which the bidirectional associations between development and relationships are emphasized as important processes in understanding behavior and functioning. From a developmental perspective, we consider the individual and environmental contributions to development, and focus on how the transactional nature of these contributions changes and grows over time throughout childhood, adolescence, and adulthood [4]. From this perspective, developmental experiences are seen as contributing to cascading trauma and violence in relationships and future substance use, given their impact on neurological and relational mechanisms. In working with substance using women and their children, we recognize that negative developmental experiences must be attended to across the lifespan to promote optimal neurological and relational development. From a relational perspective, we consider development—growth within the individual, the environment, and within and across systems—as coming about through relationships with others [5,6]. Thus, the developmental-relational approach places individuals’ behaviors (e.g., substance use) within a larger context that includes an understanding of their history and ongoing development over time, and a focus on how behavior is shaped through relationships within the broader systemic context.

This approach has been established through our research and clinical understanding of women attending a community-based prevention and early intervention program in Canada called Breaking the Cycle (BTC) [7,8]. Since 1995, Breaking the Cycle has provided comprehensive, integrated supports for mothers who are struggling with substance use issues, and their young children aged 0-6 years. Programming at BTC is directed towards women, their children, and the mother-child relationship. Over the past 25 years, we have come to understand that the vast majority of women who struggle with substance use issues in their adult lives have been traumatized in relationships since early childhood and across development. The lifelong struggle with trauma in relationships is often part of the experience faced by women with substance use issues. A developmental-relational approach can be used to more fully understand this link, as follows. (1) Experiences of interpersonal violence can be viewed as disruptions to normative developmental processes across the lifespan that can create and perpetuate lifelong trauma; (2) Interpersonal violence can begin in early childhood, including experiences of child maltreatment and neglect, and have enduring and compounding impacts, often continuing into adolescence and adulthood [9]; (3) Experiences of trauma in relationships can also involve witnessing violence between parents or caregivers, being manipulated by one caregiver to abuse the other, experiencing the aftermath of violence against a caregiver, and suffering the consequences of financial abuse, among others [10,11,12]; (4) Early traumatic experiences can also include household dysfunction, including conflictual parental divorce or separation, parental incarceration, as well as living with a parent experiencing mental health or substance use issues [13,14]. These adverse childhood experiences (ACEs) are consistently shown to relate to poor mental and physical health outcomes and wellbeing in adulthood [14,15,16]. All of these experiences are relational in nature, in that they disrupt positive bonds between caregivers and children, they damage a child’s sense of safety in relationships, and they disrupt the development of secure attachment between children and their parents [17,18]. These disruptions can have long-term consequences across development and into adulthood [19,20]. As such, it is essential to understand developmental experiences of trauma in relationships as pervading beyond early childhood, contributing to a cascade of trauma and violence in relationships given their impact on adult life, including difficulties forming healthy relationships, difficulties in parenting, ineffective coping strategies, and problematic substance use. It is also vital to consider the potential underlying neurological and relational mechanisms that may contribute to these cascading effects.

These links are evident in our work with women who struggle with substance use at BTC. Women’s initiation of substance use, problematic substance use, use of substances to cope, and inability or difficulty abstaining from substance use stem from the lifelong trauma of adverse relational experiences and their impact on development. For instance, in a sample of 160 women who had substance use issues and received services at BTC, the majority reported experiences of interpersonal violence in childhood (see [21] for a full description of the sample and methodology). Specifically, 88% of women reported a history of physical abuse, with almost half of those women (49%) reporting that the abuse began when they were 10 years or younger (see Table 1). Eighty-nine percent of women reported a history of emotional abuse, with over half of those women reporting that the abuse started before adolescence (12 years or younger). Finally, 76% of women reported a history of sexual abuse, with almost a quarter (22%) of those women reporting that the abuse began when they were five years old or younger. Almost half (43%) of women had involvement with the child welfare system when they were children.

The overwhelming majority of women at BTC have used or are currently using alcohol, and many reported that their alcohol use began at very young ages: 19% of women reported first using alcohol when they were 10 years old or younger (in the same sample of 160 women; [21]). Problematic alcohol use also began early: 7% reported that problematic use began in childhood, and an additional 56% reported that problematic use began in adolescence. Women also reported that they started to use other substances at very young ages (12 or younger), with 24% of those who used reporting early cannabis and 6% reporting early cocaine or crack cocaine use. Of the women who used, many reported that their use of these substances became problematic in adolescence (prior to age 19): 77% cannabis, 74% nicotine, 66% hallucinogens, 64% amphetamines, 43% barbiturates, 39% cocaine, 38% heroin, and 27% crack cocaine. As girls and teenagers, these women began using substances as a means of coping with the relational trauma that they were experiencing and/or had experienced. One BTC woman talked about her history of physical abuse at the hands of her mother, who was also a substance user:

All of the things I witnessed at home really affected me in my early teenage years…and at that point I became addicted myself. And so, even though I kind of had a realization that I was following in my mom’s footsteps, I wasn’t really able to do anything about it, and my own cycle of addiction kind of took over at that point.

[22] (p. 98)

Another woman discussed how her early experiences of violence and trauma had lifelong consequences on her patterns of thought and behavior.

*It creates a lifetime of fear because you’ve spent a lifetime like that, walking on eggshells, not knowing…just expected to duck the next blow…It’s something that’s been one of the hardest things in my life to challenge and attempt to change, because it’s something that I’ve been formed like…I have, you know, severe reactionary issues when it comes to safety, and conversely overreactive sense of safety*.

[23] (p. 22)

Our research has also identified the developmental pathways leading to continued problematic substance use. These pathways have been discussed as dynamic cascades or developmental cascades: early risk factors increase exposure to more risk processes that develop across the lifespan [24,25,26]. From a developmental-relational perspective, we have begun to understand how early experiences of interpersonal violence can cascade to impact and impede development across development and into adulthood. Without healthy relationships, relationship capacity is delayed and relationship perspectives are skewed (e.g., women learn to expect violence as a part of close relationships; see Section 2). As one BTC woman reported:

There was all this violence in our house, and I thought that was normal, and I thought that’s what I was supposed to be growing up. And I was receiving violence from whomever, and I just let that happen…

[27] (p. 15)

The continued impact of these relationship challenges is also evident in women’s ongoing experiences of violence, with 14% of women reporting that their current partner relationships have been abusive, and 13% of women reporting that their past partner relationships were violent (see [21] for sample details). Other close relationships appear to be impacted as well, as many women reported little to no contact with their families of origin (little to no contact with mothers, 31%; and fathers, 47%), or reported difficult and/or abusive relationships with their mothers (25%) and fathers (9%). One BTC woman talked about the effect of violence in her family:

[It] de-sensitized you a little bit…my parents were so abusive towards each other, and there was no respect or love or affection, and there was always turmoil, turmoil, turmoil – we were moving, there was fighting, there was police, there was violence – that I found out even as an adult, because that was so normal for me, if my life was going along smoothly and calmly, it’s like unfamiliar so I create this chaos, this craziness, because that feels more comfortable to me.

[27] (p. 16)

Through the work conducted at BTC, it is also evident that an intervention focusing on supporting healthy relationships can help to decrease women’s problematic use of substances. For instance, in a comparison of BTC to a standard integrated treatment program that included a focus on addiction treatment but did not focus explicitly on supporting and enhancing relationship capacity, it was found that women attending BTC had improvements in relationship capacity, mental health symptoms, as well as addiction severity [28]. Indeed, improvements in relationship capacity among women at BTC further predicted decreases in addiction severity, even accounting for other improvements, including social support, mental health, and abstinence self-efficacy. In another study, it was found that the duration of service use at BTC was associated with improvements in women’s substance use (as well as improvements in the parent-child relationship) [21]. Further, the earlier that woman began the relationship-based intervention (i.e., during pregnancy as opposed to postnatally), the more positive the outcomes. These studies provide further support for the critical link between relationship capacity and substance use issues, as evidenced by improvements based on attending a relationship-based and trauma-informed intervention program.

It should be noted that there are many co-occurring factors that compound the life challenges of women with substance use issues; these factors include poverty, low educational attainment, unstable housing, criminal involvement, and mental health difficulties (often untreated). Women at BTC reported high levels of depression and anxiety and a lack of social support from both family and friends [7]. These factors, as well as a host of other factors, are implicated in the complex interplay of, and challenges associated with, problematic substance use for women, particularly in the context of parenting. Though we won’t address these factors in detail here, they are often present and play a critical role in women’s continued substance use. Thus, we acknowledge the impact of these additional factors, but focus on using a developmental-relational approach to elucidate the role of prior trauma and experiences of relational violence across the lifespan in our understanding of women’s substance use.

## 3. Origins of Substance Use in Women Exposed to IPV

Research offers converging evidence that exposure to IPV in childhood and across development contributes to future substance use issues. Robust effects are found specifically for females within the literature, with substance use issues persisting into middle adulthood for only female (and not male) victims of childhood maltreatment [29,30,31,32] and physical abuse [33]. Although there are a few studies on the mechanisms of the intergenerational pathway from IPV in childhood to subsequent substance use issues, several processes have been proposed. The disruptive effects of early experiences of IPV on psychosocial functioning, the stress response system, and the limbic system may lead to heightened risk-taking behaviors, such as the use of substances [34,35]. Substance use may also serve as an external mechanism to cope with, or escape from, the negative effects of trauma across development [36]. Several studies have indicated that maltreatment may result in greater risk for the development of internalizing symptoms in females than in males (e.g., [37,38,39,40]). This differential risk could account, in part, for the higher incidence of internalizing problems in females relative to males (e.g., [41,42]). Therefore, as an external coping mechanism, substance use is thought to be particularly notable in women given that they may be more prone than men to internalizing symptoms due to early experiences of IPV, which can elicit self-destructive behaviors (i.e., substance use) [43]. Because substance use does not directly address the negative effects of trauma, the need for substances may persist or increase over time, thus heightening the risk for substance use issues and dependence [30]. Given that women exposed to early IPV are also more likely to have low self-esteem or low perceived self-efficacy, substance use issues have been proposed as a means through which they enhance their self-esteem [30]. Chronic substance use may also arise from these women’s low perceived self-efficacy in regards to maintaining abstinence [36]. In addition, substance use may be a means through which women are able to reduce feelings of isolation and loneliness, gain control over negative experiences, or engage in self-destructive behavior [43]. Externalizing behaviors, antisocial behaviors, and abuse-related posttraumatic stress disorder (PTSD) may mediate the relation between early experiences of IPV and future substance use issues [30]. In the following sections, we discuss the mechanisms that appear most significant in leading women to substance use within a developmental-relational perspective.

### 3.1. IPV and Neurological Development

Exposure to IPV can negatively impact neurological development, affecting physiological mechanisms, brain structure and functioning, as well as overall neuropsychological development. Although neurological development is most vulnerable to the effects of IPV during early childhood, these detrimental effects persist across the lifespan. Impairments in neurological development impact other developmental domains, including physical, cognitive, and social-emotional development. The literature highlighted in this section predominately captures studies on male and female victims of childhood maltreatment; however, some studies are specific to females with histories of childhood maltreatment or intimate partner violence (e.g., [44,45]).

#### 3.1.1. Physiological Mechanisms

Exposure to IPV can interfere with the sympathetic nervous system and the hypothalamic-pituitary-adrenal (HPA) axis. Exposure to IPV can induce chronic psychological stress that results in repeated activation of the HPA axis and subsequent HPA axis dysfunction [44,46,47]. Children exposed to IPV often have elevated baseline cortisol (stress hormone) levels, as well as a faster increase and slower decline of cortisol following stress exposures [48]. At chronically elevated levels, cortisol can have neurotoxic effects on “nonessential” brain regions during the stress response [44]. Neurotoxic effects have subsequent consequences on brain structure and functioning [49], which can persist into adulthood [44,46]. Chronic cortisol elevation also leads to increased arousal, anxiety, aggression, hypervigilance, sympathetic nervous system stimulation, depression, and PTSD [50].

#### 3.1.2. Brain Structure and Functioning

Many areas of the brain undergo neurobiological changes upon exposure to chronic stress via IPV across development. Research has identified the effects of IPV on brain structure and functioning in the midbrain, sensory cortices and fiber tracts, corpus callosum, and dopaminergic reward circuit (e.g., [51]). There is also a substantial body of research on the structural and functional effects of IPV on the stress response system through the HPA axis, which is expanded upon below.

The plasticity of the fetal, infant, and early childhood brain creates a heightened sensitivity to chemical influences of chronic stress exposure [52]. Many glucocorticoid receptors exist within the amygdala, hippocampus, and prefrontal cortex (PFC), which are part of a network of connected regions involved in the stress response. Exposure to early stressful experiences alters the size and neuronal architecture of these regions, contributing to functional differences in learning, memory, and executive functioning [46,53]. Chronic exposure to stress is associated with overactivity in the amygdala and orbitofrontal cortex, as well as the loss of neurons and neuronal connections in the hippocampus and medial PFC [53]. Functionally, these structural changes result in more fear and anxiety due to the hyperactivation of the amygdala, alongside lower higher-order PFC control [54].

The hippocampus is involved in the HPA axis and modulates cortisol levels; however, chronic stress diminishes this capacity due to hippocampal volume loss, which is linked to memory and mood-related impairments [53,54]. Chronic stress exposure can lead to impairments in memory encoding and contextual learning, which are vital for discriminating conditions of danger from safety [44,53]. Decreased neuronal volume in the PFC impairs executive and cognitive functioning; the loss of neuronal connections between the hippocampus and the PFC hinders the PFC’s regulation of heightened cortisol levels and the regulation of autonomic balance between sympathetic and parasympathetic nervous system responses [46,53]. Chronic stress also induces architectural and connection changes within and between the hippocampus, PFC, and amygdala, potentially contributing to variability in stress-responsiveness [55]. These structural changes can functionally heighten reactivity to mild levels of stress and impair coping abilities during future stress both in childhood and across the lifespan (e.g., [52]).

#### 3.1.3. Neuropsychological Development

Although research has only begun to address the structural and related functional impairments in brain development due to exposure to IPV, there is a substantial body of research on the resulting neuropsychological impairments in executive functioning and emotion regulation. Executive functioning enables flexible, context-appropriate, goal-oriented emotional and behavioral responses and is largely localized in the PFC [56]. Exposure to IPV during childhood is associated with the development of impairments in executive functioning processes, which, in turn, are associated with increased risk for PTSD and depression [46]. Deficits in executive functioning due to trauma in relationships may accumulate across development, persisting through adolescence and into adulthood; more pronounced deficits have been found with an earlier onset of trauma (e.g., [57]). Deficits in executive functioning pose heightened risk for future substance use issues; therefore, executive functioning deficits may represent a mechanism in the intergenerational pathway between IPV and substance use [58].

Differences in the structure, function, and connectivity of prefrontal regions underlying executive functioning processes are also associated with impairments in emotion regulation in adults with PTSD due to IPV [45,59]. Emotion regulation involves strategies to manage cognitive, behavioral, and physiological responses to emotions [46] and is largely localized to the anterior cingulate cortex within the PFC [44]. Children exposed to IPV often have persisting deficits in emotion regulation, including attentional biases to negative or threatening stimuli, trouble recognizing emotions, and difficulty effectively modulating or reappraising distress [46]. Problems with emotion regulation are also correlated with lower levels of social competence, difficulties with peer relationships, aggressiveness, and disruptive behaviors that can impact functioning into adulthood [47]. Additionally, emotion dysregulation contributes to mental health problems, including PTSD and depression [60]. Children exposed to IPV may struggle with emotional awareness, understanding, and regulation because such capacities are developed, in part, through interactions with supportive caregivers and adults (e.g., [61]). Children who experience IPV often receive less positive modeling of emotional labeling, expression, and regulation behaviors, which leads to deficits in appropriate emotion regulation capacities [46]. Exposure to childhood IPV is linked to emotion regulation deficits across the lifespan; however, emotion regulation is most impacted by chronic trauma in early development [46]. Given that deficits in emotion regulation pose heightened risk for future substance use issues, emotion dysregulation may represent a critical mechanism in the pathway between IPV and substance use [62,63].

### 3.2. IPV and the Development of Relational Capacity

Exposure to IPV can negatively impact the development of relational capacity across various levels, affecting attachment, internal working models, and subsequent interpersonal relationships. The impact of IPV on the development of relational capacity contributes to future substance use issues. In fact, the link between difficulties in relationships and substance use may be particularly strong for women. Relationships are important to women, and women who use substances may have less social support and are more likely than men to have important people in their lives who also struggle with substance use issues (e.g., families of origin, partners) [64,65,66,67]. Further, male partners with substance use issues may be resistant to and unsupportive of their female partners’ attempts to access treatment [68]; women may, therefore, be hesitant and fear damaging these relationships by engaging in substance use treatment. Thus, women’s initiation and continuation of substance use (including relapse) may often occur in the context of relationships, or as a result of challenges in relationships. We will describe the pathways through which early and enduring experiences of violence in relationships can affect ongoing relationship challenges into adulthood, which in turn can impact women’s substance use.

#### 3.2.1. Attachment

Attachment theory postulates that children are predisposed to seek and sustain relationships that satisfy their intrinsic need for security [69]. The failure to develop secure attachment reverberates across the lifespan in the form of difficulties with relationships, self-esteem, and the regulation of emotions and impulses [70]. Predictable, sensitive, and responsive caregiving during times of stress is crucial for healthy child development [47]; however, for children exposed to IPV, chronic stress often occurs within the context of the caregiving relationship. Research has consistently demonstrated that maltreated children have higher rates of insecure attachment, namely disorganized attachment, relative to non-maltreated children, even when compared to other high-risk children (e.g., [71]). Similarly, maltreated children have been consistently found to be at heightened risk for future substance use issues [72,73,74], with effects persisting into middle adulthood for females only [29,30,31,32]. Children classified as having disorganized attachment often vacillate between avoidant and anxious parent-child behaviors due to conflicting and unpredictable caregiver responses; these children often have poor outcomes across many domains, including lower academic achievement and self-esteem, poor peer interactions, atypical classroom behaviors, cognitive immaturity, and externalizing behavioral concerns (e.g., [70]). Disorganized attachment is further associated with poor mental health outcomes in adulthood, including borderline personality disorder and dissociative identity disorder [75]. Although much research has indicated the relationship between maltreatment and future substance use issues, a growing body of literature has begun to address attachment as a key mechanism in this relationship [74]. Attachment insecurity with caregivers poses a heightened risk for future substance use issues in adolescence and adulthood [76,77,78,79,80]. There is also strong evidence for the temporal precedent of attachment issues, with insecure attachment predating the onset or increased use of substances across time [81].

#### 3.2.2. Internal Working Models

The characteristic patterns of caregivers’ responses to children’s expression of attachment behavior accumulate over time [82]. These patterns are organized into schematic cognitive representations of the parent-child relationship, theorized as internal working models of attachment [69,83]. Children use their internal working models of attachment to perceive and appraise attachment-related information and to plan future action [83]. Based on the internal working model of attachment, children develop expectations about the self and others: the self as worthy or unworthy of care and protection and others as available or unavailable to provide care and protection when needed [69]. Children exposed to IPV develop negative models of themselves as unworthy of care and protection, and models of their caregivers as rejecting and unreliable [71]. Although internal working models of attachment develop across the lifespan alongside changes in cognitive capacity and attachment relationships, the models show great stability throughout life [84].

Children who experience IPV early in life may transfer their negative internal working models of attachment to future relationships, thus expecting the same abuse in adult relationships and viewing such abuse as normative [85,86]. Therefore, according to attachment theories, IPV in early caregiving relationships initiates a developmental cascade in which insecure attachments continue to occur across the lifespan, due to existing insecure internal working models [87]. In addition to impacting attachment in future relationships, internal working models contribute to one’s ability to regulate emotions autonomously in the absence of an attachment figure [77]. As discussed above, deficits in emotion regulation contribute to future substance use issues, thus representing a mechanism in the pathway between IPV and substance use given the impact on attachment and internal working models [62,63]. Furthermore, IPV and insecure attachment are more common in children whose parents also experienced IPV and insecure attachment [88]. Therefore, there is intergenerational transmission of both attachment styles and IPV (e.g., [89,90,91]). Attachment styles and the development of internal working models have a putative mediating role in the intergenerational transmission of IPV and subsequent substance use issues, given that IPV becomes a frame through which people come to understand relationships, and substance use becomes a means through which people regulate emotions in the absence of secure attachment and positive internal working models [76,77,87]. This may be particularly problematic for women, given the importance women place on relationships and the lack of social support that substance using women often face (in comparison to men) [64,65].

### 3.3. A Model of IPV and Substance Use Across the Lifespan

**Figure 1:**
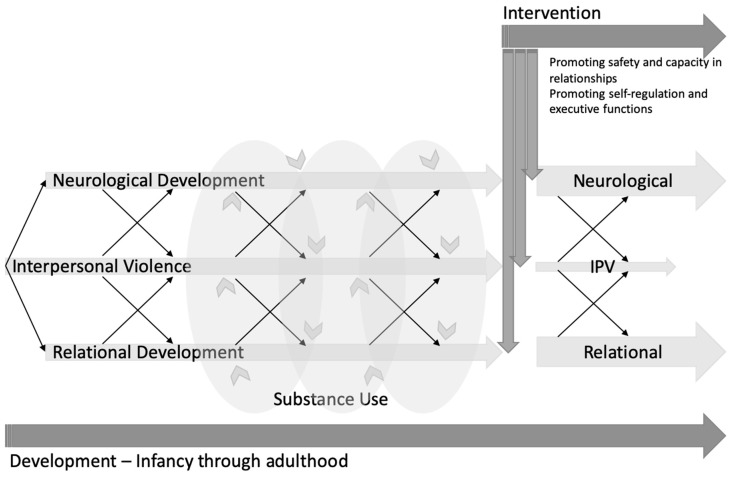
A model of interpersonal violence (IPV) and substance use (SU) across the lifespan, highlighting the bidirectional effects of IPV on neurological and relational development.

Overall, early and enduring experiences of IPV negatively impact neurological development, namely physiological mechanisms, brain structure and functioning, as well as neuropsychological development (i.e., executive functioning and emotion regulation). Experiences of IPV across development are traumatic and disrupt the development of relational capacity, specifically attachment and internal working models that affect relationships characterized by IPV across the lifespan [71]. IPV negatively impacts executive functioning [46] and emotion regulation [44,46,47], which are also impaired through substance use issues [92]. There is a strong relationship between childhood IPV and future substance use issues [30], thus compounding the negative effects that both factors have on executive functioning and emotion regulation. These neuropsychological deficits interact to divert development onto a pathway toward unhealthy relationships. At the same time, these deficits elicit substance use as a necessary means of coping (see Figure 1 for an illustrative model).

## 4. Conclusions

In this review paper, we have outlined how a developmental-relational approach helps us understand the link between women’s experiences of violence in relationships across development and later substance use issues. Potential mechanisms that underly this pathway include impairments to neurological development, namely physiological mechanisms, brain structure and functioning, and neuropsychological development (i.e., executive functioning and emotion regulation), as well as impairment to the development of relational capacity (i.e., attachment and internal working models). Though there is a paucity of research specifically examining differences in these links between women and men (particularly regarding the neurological effects of childhood maltreatment), there is some evidence that early experiences of IPV may be particularly problematic for women and may lead to enduring substance use into adulthood. For instance, women may internalize trauma and turn to substances as a coping mechanism more so than men [43], and women’s initiation and continuation of substance use seems to occur often in the context of relationships [66,67], which are negatively impacted by early experiences of IPV.

Through this review, we have highlighted that IPV is a lifelong disrupting force on women’s neurological development and capacity for relationships, which can lead women to use substances, as well as to further developmental and relational impairments. These factors can perpetuate the pathway of IPV across the lifespan, while reinforcing substance use as a necessary means of coping. A developmental-relational approach to understanding substance use, therefore, has important implications for clinical practice. In a recent review, we outlined specific strategies that can be used to promote women’s self-regulation and executive functions (e.g., supporting time management through reminder phone calls and predictable appointments), and promoting safety and capacity in relationships (e.g., staff training in trauma-informed practice) [93]. Attention should also be paid to strategies that may be uniquely important for women (e.g., including a buzzer entry system; not allowing male partners in the center) and mothers (e.g., providing child-minding and child development programming in the same program location). As such, by using a developmental-relational approach to understand women’s pathways to substance use, we can begin to engage in a process of reparation and reintegration for women whose neurological development, sense of self, and capacity to form relationships has been significantly impacted by experiences of violence in relationships.

## Figures and Tables

**Table 1:** Percentage of Women Reporting Histories of Abuse Across Childhood.

Onset of Abuse	Physical Abuse (%)	Emotional Abuse (%)	Sexual Abuse (%)
Percentage of women reporting histories of abuse (total)	88	89	76
Onset (among women who reported abuse)			
“As long as I can remember”	5	10	0
Early childhood	9	7	22
Childhood	35	33	20
Late childhood	5	4	6
Early adolescence	6	6	13
Adolescence	15	24	12
Late adolescence	3	1	8
Adulthood	22	15	19

Early childhood = 0–5 years. Childhood = 6–10 years. Late childhood = 11–12 years. Early adolescence = 13–14 years. Adolescence = 15–16 years. Late adolescence = 17–18 years. Adulthood = 19 years or older.
